# Association of the Extension for Community Healthcare Outcomes Project With Use of Direct-Acting Antiviral Treatment Among US Adults With Hepatitis C

**DOI:** 10.1001/jamanetworkopen.2021.15523

**Published:** 2021-07-02

**Authors:** Linh Tran, Roger Feldman, Thomas Riley, Jeah Jung

**Affiliations:** 1 Department of Health Policy and Administration, Pennsylvania State University College of Health and Human Development, University Park, State College; 2 Division of Health Policy and Management, University of Minnesota School of Public Health, Minneapolis; 3 Department of Medicine, Pennsylvania State University College of Medicine, Hershey

## Abstract

**Importance:**

Direct-acting antiviral (DAA) medications are highly effective in treating hepatitis C virus (HCV) infection. However, use of DAAs in rural and underserved areas is low owing to limited access to specialist physicians with experience in care of HCV infection. Project ECHO (Extension for Community Healthcare Outcomes) is a distance education model that trains primary care physicians to improve access to care for underserved populations with complex diseases such as HCV infection. Evidence on whether Project ECHO is associated with increased DAA use is limited.

**Objective:**

To examine the association between Project ECHO and use of DAA treatment in patients with HCV infection.

**Design, Setting, and Participants:**

This cohort study used data from Medicare beneficiaries who newly sought care for HCV infection between January 1, 2014, and December 31, 2017. Data were analyzed between September and December 2020.

**Exposures:**

Project ECHO.

**Main Outcomes and Measures:**

Use of DAA treatment. Discrete-time hazard models with state and year fixed effects were used to examine the association between Project ECHO and DAA use in rural areas and areas with low specialist density.

**Results:**

A total of 267 908 patients (mean [SD] age, 60.7 [11.5] years; 57.9% male; 66.6% White patients) were included in the analysis. For every 100 clinicians attending a Project ECHO training, the odds of DAA treatment initiation among patients with HCV infection increased by 9% (adjusted odds ratio [OR], 1.09; 95% CI, 1.07-1.11; *P* < .001) in nonrural areas with specialist density equaling 0. The association between DAA use and Project ECHO was stronger in areas with lower vs higher specialist density. For every additional 100 Project ECHO participants, the odds of DAA use decreased by 1% as specialist density in the area increased (adjusted OR, 0.99; 95% CI, 0.98-1.00; *P* = .03). There was no association between Project ECHO and the odds of receiving DAAs among patients in rural vs urban areas (adjusted OR, 1.01; 95% CI, 0.99-1.02; *P* = .49).

**Conclusions and Relevance:**

In this cohort study, implementation of Project ECHO was associated with increased DAA use in areas with few specialist physicians, suggesting that Project ECHO may enhance access to DAA treatment through expanding the capacity of primary care physicians to treat HCV infection, especially in underserved areas.

## Key Points

**Question:**

Is Project ECHO (Extension for Community Healthcare Outcomes), a distance education model that trains primary care physicians to improve access to care for underserved populations, associated with increased use of direct-acting antiviral treatment for hepatitis C virus (HCV) infection?

**Findings:**

In this cohort study of 267 908 patients, for every 100 additional clinicians who attended a Project ECHO training session, the odds that a patient with HCV infection would receive a direct-acting antiviral agent increased by 9%. Project ECHO was associated with increased direct-acting antiviral agent use in areas with few specialist physicians compared with areas with higher specialist density.

**Meaning:**

These results suggest that Project ECHO may be a promising strategy to improve access and reduce barriers to treatment of HCV infection, especially in underserved areas.

## Teaser

This cohort study uses data from Medicare beneficiaries to assess the association between Project ECHO (Extension for Community Healthcare Outcomes) and increased use of hepatitis C virus treatment, particularly in resource-limited areas.

## Introduction

Hepatitis C virus (HCV) infection is a public health concern, with approximately 2.7 to 3.9 million people in the US having chronic infection.^1^ Untreated HCV infection can result in serious health problems, such as cirrhosis, hepatocellular cancer, and liver damage.^2^ Recently developed second-generation direct-acting antiviral (DAA) drugs have cure rates greater than 90%, which is higher than the 50% cure rate with former interferon-based treatments.^3^ In addition, DAAs are easy to administer (1 pill per day), require a short treatment period (8-12 weeks), and have few adverse effects. Direct-acting antiviral therapy is associated with a 49% to 68% reduction in HCV all-cause mortality.^3,4^ The availability of DAAs has therefore given hope that HCV infection can be eliminated.^5^ The World Health Organization has set a goal of reducing the incidence of HCV infection by 80% by 2030 and has recommended treating all adults with chronic HCV infection with DAAs.^6,7^

Despite the high cure rates of DAAs, use of DAA therapy is low at less than 50% between 2014 and 2016 (the first DAA, sofosbuvir, was approved on December 1, 2013).^1,8,9,10,11^ Substantial rural-urban variation in receipt of DAA treatment exists.^1^ The main barriers to providing DAAs to rural populations are the high cost of DAAs, lack of specialists with experience in care for HCV infection, and distance and travel time.^12,13,14^ For example, patients from remote areas may wait up to 6 months and travel up to 250 miles for an appointment.^13^

Limited access to specialty services at community-based health centers is another reason why patients fail to access treatment for HCV infection.^13^ Most primary care physicians (PCPs) do not feel comfortable offering treatment for HCV infection owing to a lack of training, especially in resource-limited settings, and thus they often refer patients with HCV infection to specialty care.^13,14^

In response to the need for specialty services in rural and underserved areas, Project ECHO (Extension for Community Healthcare Outcomes), a telementoring program, was launched in 2003 by the University of New Mexico Health Sciences Center.^15^ Project ECHO aims to reduce health disparities and expand access to medical care for underserved populations with complex conditions, such as HCV infection.^15,16^ Through the use of video conferencing technology, Project ECHO adopts a hub-and-spoke approach to connect a specialist team (the hub) with PCPs or other health care professionals such as community health workers (the spokes).^13,15,16^ Primary care physicians can participate in Project ECHO at no cost.^17^ The only associated costs are those for technology equipment (if needed) and time away from the clinic.^17^ Many Project ECHO sessions are offered in the early morning or during lunch hours to minimize the time away from direct patient care.^17^ After participating in Project ECHO, PCPs can develop treatment plans and provide specialized services for patients in their communities based on recommendations from the specialists at the hub.^13,15,16^

Project ECHO may be a promising approach to reduce the need for specialist referrals. By providing training and support for PCPs in prescribing DAAs, Project ECHO makes curative treatment accessible to patients living in underserved areas.^18,19,20^ Project ECHO–trained PCPs provide safe and effective treatment for HCV infection in rural and remote areas with guidance and consultation from specialists.^13^ The quality of care for HCV infection delivered by ECHO-trained PCPs was reported to be comparable to that of specialists.^13,21^

Evidence on the association of Project ECHO with DAA use is limited. One study found that Project ECHO was associated with reduced urban-rural disparities in DAA uptake in 2 states.^12^ However, the results might not generalize beyond those 2 states. Thus, we extended the prior research by examining the association between Project ECHO and DAAs using national data from Medicare, a federal health insurance program for older adults and those with disabilities in the US. Because many HCV-infected individuals in the US were born between 1945 and 1964,^22^ Medicare covers a group with a high prevalence of HCV infection and thus has a substantial role in care for HCV infection.

In addition to using national data, our study examined the association of Project ECHO with access to treatment for HCV infection in underserved areas with low specialist physician density. Although some states have discontinued their Project ECHO, trained PCPs may still be able to use medical knowledge gained from past clinic sessions to deliver high-quality care to patients with HCV infection in their own communities. Therefore, we measured Project ECHO by the cumulative number of attendees in HCV-specific ECHO sessions since its implementation in each state. We examined the association between Project ECHO and DAA treatment in a national cohort of Medicare beneficiaries with HCV infection and evaluated whether that association was larger in underserved and rural areas.

## Methods

### Data

This cohort study used data from patients with HCV infection identified using Medicare claims for inpatient, skilled nursing facility, hospital outpatient, and physician services from January 1, 2013, to December 31, 2017 (2013 data were used only to ensure that patients did not seek care for HCV infection in 2013). The initiation of DAAs was identified from Medicare Part D data from January 1, 2014, to December 31, 2017. The institutional review board of the Pennsylvania State University approved the study, and a waiver for informed consent was provided owing to the use of the existing administrative data. This study followed the Strengthening the Reporting of Observational Studies in Epidemiology (STROBE) reporting guideline.

We requested information on the annual number of physicians attending HCV-specific Project ECHO clinic sessions from 2006 to 2017 from the University of New Mexico Health Sciences Center (data before 2006 were not available). Additional information on Project ECHO is available elesewhere.^23^ We obtained patient demographic characteristics, health risks, and death dates from Master Beneficiary Summary Files; state-level total and rural population aged 45 years or older from the American Community Survey; county-level specialist density and number of PCPs from the Area Health Resources Files; and ZIP-level rurality of residence based on Rural-Urban Commuting Area codes.^24^

### Study Sample

The study sample included fee-for-service Medicare beneficiaries who newly sought care for HCV infection between January 1, 2014, and December 31, 2017, after a 1-year washout period. This criterion identified patients who were at a relatively similar stage of seeking care for HCV infection. An index date for each beneficiary was the date of their first HCV-related claim after a 1-year period with no HCV-related claims. Patients with HCV infection were identified by the standard algorithm used by the Centers for Medicare & Medicaid Services^25^: at least 1 inpatient or skilled nursing facility claim or at least 2 hospital outpatient or physician claims for HCV infection in a given year. Hepatitis C virus–related claims were identified using *International Classification of Diseases, Ninth Revision* codes 070.44, 070.54, 070.70, 070.71 or *International Statistical Classification of Diseases and Related Health Problems, Tenth Revision* codes B18.2, B19.20, B19.21.

Although the first DAA (sofosbuvir) was approved by the US Food and Drug Administration in late 2013, patients were required to take sofosbuvir and ribavirin with pegylated interferon. Interferon-based regimens were usually provided by specialists because of frequent adverse effects, suboptimal response rates, and long durations of treatment.^19^ Thus, patients who took sofosbuvir with interferon were excluded from our analysis. We excluded patients residing in New Mexico because ECHO was first launched in New Mexico, and individuals from this state account for almost half of the cumulative number of attendees. This exclusion helped prevent the results from being overly affected by a single state. We included patients from New Mexico in a sensitivity analysis.

**Figure 1.**
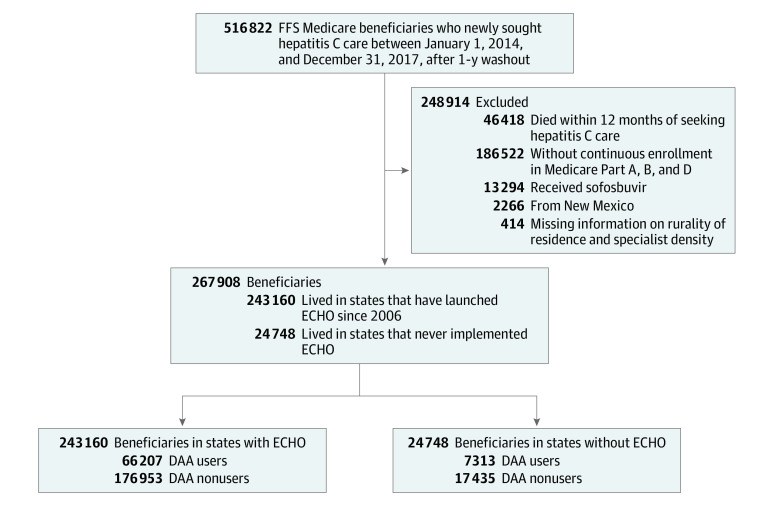
Study Sample Selection DAA, direct-acting antiviral; ECHO, Extension for Community Healthcare Outcomes; FFS, fee for service.

We excluded patients who died within 12 months after the index date because DAAs are not recommended for patients with less than 1-year life expectancy.^26^ We excluded patients who did not have continuous coverage in Medicare Part A (hospital and post–acute care), Part B (outpatient medical care), and Part D (outpatient prescription drugs) throughout the entire year; those who enrolled in Medicare Advantage plans; and those with missing information on ZIP code–level rurality and county-level physician density. Figure 1 presents a diagram of the study sample selection.

### DAA Use and Project ECHO

We defined DAA use as filling at least 1 prescription for 1 of the following DAAs between 2014 and 2017: elbasvir and grazoprevir; ledipasvir and sofosbuvir; ombitasvir, paritaprevir, and ritonavir plus dasabuvir, sofosbuvir, and velpatasvir; sofosbuvir, velpatasvir, and voxilaprevir; or glecaprevir and pibrentasvir.

**Figure 2.**
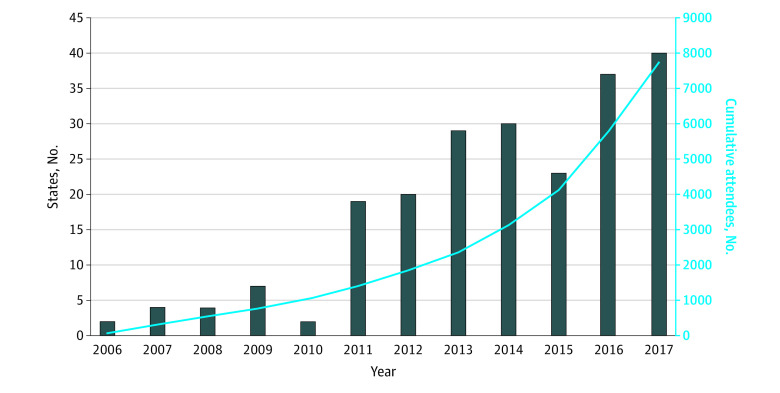
Time Trend in Adoption of the Project for Extension for Community Healthcare Outcomes (ECHO) for Care of Hepatitis C Virus (HCV) Infection States with an active Project ECHO program had at least 1 attendee in HCV sessions in a given year. The cumulative number of attendees was calculated by adding the annual numbers of attendees between 2006 and 2017.

The variable of interest was the cumulative number of attendees in HCV-specific Project ECHO sessions since its implementation in each state (Table 1). The total number of physicians attending Project ECHO clinic sessions increased over time. Project ECHO was first launched in New Mexico in 2003, but it was not widely adopted in other states until 2011 (Figure 2). By 2017, Project ECHO had expanded to 40 states with nearly 8000 trained clinicians.

**Table 1.** Extension for Community Healthcare Outcomes Cumulative Number of Attendees in Hepatitis C Sessions by State, 2014-2017

State	Attendees, No.^a^			
	2014	2015	2016	2017
Alabama	NA	NA	3	13
Arkansas	18	40	57	76
Arizona	95	137	201	256
California	60	102	244	403
Colorado	22	32	53	68
Connecticut	124	170	221	275
District of Columbia	15	15	15	15
Florida	1	1	3	4
Georgia	6	6	11	17
Hawaii	5	6	6	6
Idaho	93	124	148	179
Illinois	36	163	383	608
Indiana	NA	NA	3	9
Iowa	2	2	2	2
Kansas	NA	NA	2	8
Kentucky	7	7	7	7
Louisiana	8	14	33	44
Maine	NA	NA	3	11
Maryland	3	3	5	7
Massachusetts	21	29	64	117
Michigan	1	1	3	10
Minnesota	25	31	43	57
Mississippi	NA	NA		1
Missouri	6	6	37	94
Montana	74	114	156	195
Nebraska	3	5	6	7
Nevada	2	5	8	11
New Hampshire	NA	NA		1
New Jersey	15	15	17	17
New Mexico	2066	2308	2658	3008
New York	13	13	46	92
North Carolina	1	1	1	1
North Dakota	3	7	21	31
Ohio	4	4	6	8
Oklahoma	24	61	165	257
Oregon	41	68	95	138
Pennsylvania	7	17	23	23
Rhode Island	NA	NA	NA	2
South Carolina	NA	NA	NA	3
South Dakota	7	7	16	22
Tennessee	1	1	3	10
Texas	85	178	286	380
Utah	91	142	196	275
Virginia	25	25	25	26
Washington	101	223	454	746
West Virginia	NA	NA	74	134
Wyoming	30	45	63	78

Abbreviation: NA, not available.

^a^ The value of cumulative number of attendees in hepatitis C sessions depended on when the beneficiary entered the study. For example, if 1 patient entered the study in 2014 and another patient in the same state entered in 2017, they would have different values for the exposure of interest.

To assess the association between Project ECHO and DAA use in rural and underserved communities, we included interactions of the cumulative number of Project ECHO attendees with rural-urban status and specialist density. The rural-urban factor was based on Rural-Urban Commuting Area codes of the patient’s ZIP code residence.^24^ Specialist density was measured by county-level specialists per 1000 population, which was used to capture underserved areas.

### Covariates

We controlled for patients’ sex, race/ethnicity, age, and health risks measured by indicators of cirrhosis, HIV infection and AIDS, cancer, cardiac diseases, diabetes, eye diseases, kidney diseases, drug- and alcohol-related disorders, and bone diseases. We included ZIP code–level rurality and county-level specialist density and the number of PCPs. We controlled for total and rural populations aged 45 years or older in each state because HCV infection is more prevalent in persons older than 40 years.^27^ We included dummies for the patient’s state of residence and year to account for time-invariant state characteristics and annual trends that may have affected DAA use in all states. The data on DAA initiation dates and time-varying covariates were measured to the nearest year.

### Statistical Analysis

Data were analyzed from September to December 2020. We used a difference-in-differences estimator exploiting the variation in exposure to Project ECHO training by identifying changes in DAA use between 2 groups: states that implemented the program (treatment group) and those that did not (control group). We compared changes in odds of DAA use for patients in the treatment and control group using a discrete-time hazard model. Because the dependent outcome, DAA initiation, was a 1-time event and not all patients experienced the event, the discrete-time hazard model was an appropriate form of analysis. Individuals were considered at risk for DAA use when they received a diagnosis of HCV infection, and they are removed from the risk pool after initiating a DAA. Patients who did not use a DAA were censored at the end of the study or the time of death. Details of the discrete-time hazard model are described in the eAppendix in the Supplement.

Marginal effects were calculated to quantify the incremental risk associated with Project ECHO by specialist density percentile. We considered a 2-sided *P* < .05 to be significant in all analyses. Analyses were performed using SAS, version 9.4 (SAS Institute Inc) and Stata, version 15 (StataCorp LLC).

Almost half of the cumulative number of Project ECHO–trained physicians practiced in New Mexico. We included patients in New Mexico in a sensitivity analysis to check the robustness of the primary analysis results. We also performed sensitivity analysis including patients who died within 12 months from the index date. These patients were excluded from the primary analysis because DAAs are not recommended for patients with less than 1-year life expectancy.^26^ However, this exclusion might not have identified patients for whom DAA treatment was contraindicated on the basis of their life expectancy.

A third sensitivity analysis used an alternative measure for Project ECHO: a binary variable for an active Project ECHO program in a state in a given year. The association between Project ECHO and DAA use might not exist in states with too low a number of attending physicians; therefore, we considered that a state had an active ECHO program if it had at least 6 attendees participating in Project ECHO HCV clinic sessions in a given year. The detailed Project ECHO status of each state is given in eTable 1 in the Supplement. In addition to the aforementioned covariates, we controlled for number of years since Project ECHO implementation to examine the impact of the program over time.

## Results

The analysis included 267 908 patients (mean [SD] age, 60.7 [11.5] years; 57.9% male; 66.6% White patients), consisting of 243 160 patients (mean [SD] age, 61.0 [11.5] years; 58.0% male) in states that had launched Project ECHO between 2006 and 2017 and 24 748 patients (mean [SD] age, 58.0 [11.6] years; 56.9% male) in states that had never implemented Project ECHO. The relatively low proportion of women reflects the higher prevalence of HCV infection among men than among women.^28^ Patient characteristics are reported in Table 2. Patients in states that had ever implemented Project ECHO were more likely to be male, African American, and older; live in urban settings; and have comorbid conditions.

**Table 2.** Characteristics of Medicare Beneficiaries With Hepatitis C, 2014-2017

Variable	Patients^a^		
	Overall (n = 267 908)	State with a Project ECHO HCV program (n = 243 160)	State with no Project ECHO HCV program (n = 24 748)
DAA use	73 520 (27.4)	66 207 (27.2)	7313 (29.6)
Geographic characteristics			
Rural	50 943 (19.2)	42 965 (17.7)	7977 (32.2)
Specialist density, mean (SD), per 1000 population	1.1 (1.0)	1.2 (1.0)	0.9 (0.7)
Primary care physicians at county level, mean (SD), No. in 1000s	2.7 (4.5)	2.9 (4.7)	0.7 (0.9)
Total population aged 45 y or older, mean (SD), No. in 100 000s	54.6 (43.5)	58.7 (43.6)	15.1 (7.9)
Total rural population aged 45 y or older, mean (SD), No. in 100 000s	15.0 (15.9)	15.4 (16.6)	11.0 (3.7)
Sex			
Women	112 811 (42.1)	102 143 (42.0)	10 666 (43.1)
Men	155 097 (57.9)	141 033 (58.0)	14 082 (56.9)
Age, mean (SD), y	60.7 (11.5)	61.0 (11.5)	58.0 (11.6)
Race/ethnicity			
White	178 462 (66.6)	160 054 (65.8)	18 408 (74.4)
African American	66 408 (24.8)	60 872 (25.0)	5536 (22.4)
Hispanic	8902 (3.3)	8656 (3.6)	246 (1.0)
Other^b^	14 136 (5.3)	13 578 (5.6)	558 (2.3)
Clinical comorbidities			
Cirrhosis	42 396 (15.8)	38 518 (15.8)	3878 (15.7)
HIV infection or AIDS	16 711 (6.2)	15 530 (6.4)	1181 (4.8)
Cancer	36 923 (13.8)	34 177 (14.1)	2746 (11.1)
Diabetes	94 874 (35.4)	87 014 (35.8)	7860 (31.8)
Cardiac disease	207 063 (77.3)	188 663 (77.6)	18 398 (74.4)
Eye disease	43 979 (16.4)	40 521 (16.7)	3458 (13.8)
Bone disease	114 132 (42.3)	102 887 (42.3)	10 397 (42.0)
Kidney disease	87 382 (32.6)	80 268 (33.0)	7114 (28.8)
Drug- and alcohol-related disorders	148 936 (55.1)	132 645 (54.6)	14 985 (60.6)

Abbreviations: DAA, direct-acting antiviral; ECHO, Extension for Community Healthcare Outcomes; HCV, hepatitis C virus.

^a^ Data are presented as number (percentage) of patients unless otherwise specified.

^b^ Other includes Asian/Pacific Islander, American Indian, and unknown.

Table 3 presents the results from the discrete-time hazard model. Compared with states that never implemented Project ECHO, in states that implemented Project ECHO, for each additional 100 clinicians attending the program, the odds of initiating a DAA among patients with HCV infection increased by 9% (adjusted odds ratio [OR], 1.09; 95% CI, 1.07-1.11; *P* < .001) in nonrural areas with specialist density equaling 0. The interaction term between the cumulative number of attendees and specialist density showed that Project ECHO was associated with increased DAA use in counties with low specialist density (adjusted OR, 0.99; 95% CI, 0.98-1.00; *P* = .03). The marginal treatment effect of Project ECHO for DAA use was 1.21 percentage points (95% CI, 0.08-1.06 percentage points; *P* = .02) in counties with low specialist density and 0.57 percentage points (95% CI, 0.95-1.48 percentage points; *P* < .001) in counties with a high density of specialist (eTable 2 in the Supplement). The interaction term between the number of ECHO-trained health care professionals and the rural-urban indicator suggested that Project ECHO was not associated with the odds of DAA use among patients in rural vs urban areas (adjusted OR, 1.01; 95% CI, 0.99-1.02; *P* = .49).

**Table 3.** Adjusted Odds Ratios of Direct-Acting Antiviral Treatment Initiation Among 267 908 Beneficiaries^a^

Variable	Odds ratio (95% CI)	*P* value
Project ECHO program, 100 physicians attending Project ECHO	1.09 (1.07-1.11)	<.001
Interaction		
Project ECHO program × rural	1.01 (0.99-1.02)	.49
Project ECHO program × specialist density	0.99 (0.98-1.00)	.03
Geographic characteristics		
ZIP code level		
Urban	1 [Reference]	NA
Rural	1.00 (0.97-1.03)	.79
County level		
Specialist density, per 1000 population	1.01 (0.99-1.02)	.42
PCPs, No. in 1000s	1.00 (0.99-1.00)	<.001
State level		
Total population aged 45 y or older, No. in 100 000s	0.96 (0.95-0.97)	<.001
Total rural population aged 45 y or older, No. in 100 000s	0.89 (0.77-1.03)	.13
Women	0.89 (0.88-0.91)	<.001
Age	1.00 (1.00-1.00)	.18
Race/ethnicity		
White	1 [Reference]	<.001
African American	1.54 (1.51-1.58)	
Hispanic	0.91 (0.87-0.96)	
Other^b^	0.79 (0.76-0.82)	
Clinical comorbidities		
Cirrhosis	2.09 (2.05-2.13)	<.001
HIV infection or AIDS	1.08 (1.04-1.12)	
Cancer	0.81 (0.79-0.83)	
Diabetes	0.93 (0.91-0.95)	
Cardiac disease	0.73 (0.71-0.74)	
Eye disease	1.12 (1.09-1.14)	
Bone disease	0.97 (0.95-0.98)	
Kidney disease	0.60 (0.59-0.61)	
Drug- and alcohol-related disorders	0.60 (0.59-0.61)	

Abbreviations: ECHO, Extension for Community Healthcare Outcomes; NA, not available; PCP, primary care physician.

^a^ Includes 403 228 person-years.

^b^ Includes Asian/Pacific Islander, American Indian, and unknown.

Some patient factors were significantly associated with the odds of DAA initiation. Being female was associated with 10.6% lower odds of DAA initiation (adjusted OR, 0.89; 95% CI, 0.88-0.91; *P* < .001). The odds of DAA use were 54% higher among African American patients (adjusted OR, 1.54; 95% CI, 1.51-1.58; *P* < .001) compared with White patients. The presence of certain comorbidities, such as cancer (adjusted OR, 0.81; 95% CI, 0.79-0.83), diabetes (adjusted OR, 0.93; 95% CI, 0.91-0.95), cardiac disease (adjusted OR, 0.73; 95% CI, 0.71-0.74), bone disease (adjusted OR, 0.97; 95% CI, 0.95-0.98), kidney disease (adjusted OR, 0.60; 95% CI, 0.59-0.61), and drug- and alcohol-related disorders (adjusted OR, 0.60; 95% CI, 0.59-0.61) was associated with lower odds of DAA initiation (all *P* < .001). Patients with cirrhosis (adjusted OR, 2.09; 95% CI, 2.05-2.13), HIV/AIDS (adjusted OR, 1.08; 95% CI, 1.04-1.12), and eye diseases (adjusted OR, 1.12; 95% CI, 1.09-1.14) were more likely to use DAAs (all *P* < .001). Beneficiaries with cirrhosis had twice the odds of initiating a DAA than those without cirrhosis. Residing in rural areas (adjusted OR, 1.00; 95% CI, 0.97-1.03; *P* = .79) and specialist density (adjusted OR, 1.01; 95% CI, 0.99-1.02; *P* = .42) were not associated with the odds of receiving DAAs (Table 3).

The findings from the analysis including patients residing in New Mexico were similar, and in many cases identical, to those of the main analysis (eTable 3 in the Supplement). In this analysis, Project ECHO was associated with increased DAA use (adjusted OR, 1.08; 95% CI, 1.07-1.10; *P* < .001). The interaction between Project ECHO and specialty density also showed a negative association with DAA use (adjusted OR, 0.99; 95% CI, 0.99-1.00; *P* = .02). The results from the analysis including patients who died within 12 months from the index date were similar to those of the main analyses (eTable 4 in the Supplement).

The results from the analysis using a Project ECHO variable (whether the state had >5 ECHO attendees in a given year) were also similar to those of the primary analysis. Although the results suggest that Project ECHO was not associated with DAA use (adjusted OR, 1.01; 95% CI, 0.96-1.06; *P* = .98) (eTable 5 in the Supplement), Project ECHO was associated with 3% increased odds of initiating a DAA in areas with low specialist density (adjusted OR, 0.97; 95% CI, 0.95-0.99; *P* = .007).

## Discussion

In this cohort study, implementation of Project ECHO was associated with improved access to DAA treatment. An additional 100 PCPs participating in Project ECHO HCV sessions was associated with 9% increased odds of DAA use after adjusting for patient demographic characteristics, comorbidities, and geographic characteristics.

Project ECHO was associated with increased odds of DAA use in areas with few specialist physicians compared with areas with higher specialist density. Although the magnitude of the association was small, this finding suggests that Project ECHO can potentially address 1 of the major barriers to expanding access to treatment of HCV infection: specialist shortages. After attending telementoring sessions, PCPs in resource-limited settings are able to deliver specialized care for complex health conditions such as HCV infection in community-based clinics where specialist services were previously unavailable.^16^ As a result, access to DAA treatment is less restricted by limited numbers of hepatologists, gastroenterologists, and infectious diseases specialists in underserved areas. Even though we did not find an association between Project ECHO and DAA use in rural areas compared with urban areas, our results showed that the number of health care professionals attending Project ECHO HCV clinic sessions was associated with increased DAA use.

We found that the odds of DAA use were higher among African American patients compared with White patients. This is consistent with a prior study, which suggested that increased DAA use among African Americans may reflect pent-up demand for treatment for HCV infection because of poor tolerance for interferon-based therapy.^8^ The fight to eradicate HCV infection in the US has been ongoing for many years without success. Despite the recommendation of the World Health Organization to treat all adults with chronic HCV infection with DAAs,^29^ only 27% of Medicare patients with HCV infection in our study initiated an interferon-free DAA. This finding is consistent with those of previous studies of Medicare populations with HCV infection.^8,12,30^ A major reason for low DAA uptake is the insufficient numbers of physicians with experience in cases of HCV infection, especially in rural and underserved areas.^12^ However, with task-shifting treatment models such as Project ECHO, access to DAA treatment has become less limited by the shortage of specialists. Our study suggests that Project ECHO is associated with increased availability of and access to DAA therapy, particularly in resource-limited settings, moving a step closer to the HCV infection treatment target set by the World Health Organization.^6^

### Limitations

This study has limitations. First, we could not obtain information on how many patients with HCV infection were treated by rural physicians who participated in Project ECHO or the percentage of rural PCPs who attended Project ECHO HCV sessions. Thus, we may not have fully captured the role of the intervention in the care of HCV infection in rural areas. Second, health care professionals were eligible to receive continuing medical education credits if they participated in all Project ECHO HCV sessions or 70% of the sessions within a series. This may have influenced whether the physicians were prepared to provide specialized care to their patients with HCV infection. We did not have information on ECHO completion for each participant. Third, we did not know whether health care professionals trained before 2014 continued to treat patients with HCV infection when DAAs became available. These physicians may have retired, moved to other states, or died or may not have adopted use of DAAs. Therefore, we may have underestimated the association between Project ECHO and DAA use. Fourth, owing to limitations of claims data, we did not have detailed clinical information such as viral clearance; thus, some patients treated with DAAs in our analysis may not have been cured of their HCV infection. Fifth, our study was limited to fee-for-service Medicare beneficiaries; thus, the results may not apply to other populations.

## Conclusions

The results of this cohort study suggest that implementation of ECHO was associated with improved access to DAA treatment, suggesting that Project ECHO may be a promising strategy to improve access and reduce barriers to HCV treatment, especially in underserved areas. Expansion of Project ECHO may help mitigate unmet needs for DAA treatment and expand capacity of care for patients with HCV infection in resource-limited settings. More research is needed to evaluate whether Project ECHO improves access to other recommended care for patients with HCV infection.

## Notes

Supplement. **eAppendix.** Discrete-Time Hazard Analysis **eTable 1.** Status of Extension for Community Healthcare Outcomes (ECHO) Status for Hepatitis C Care by State, 2014-2017 **eTable 2.** Marginal Effects of Extension for Community Healthcare Outcomes (ECHO) on Direct-Acting Antiviral (DAA) Initiation by Specialist Density Percentile **eTable 3.** Adjusted Odd Ratios of Direct-Acting Antiviral (DAA) Initiation (Including Patients from New Mexico) **eTable 4.** Adjusted Odd Ratios of Direct-Acting Antiviral (DAA) Initiation (Including Patients Died within 12 Months from The Index Date*) **eTable 5.** Adjusted Odd Ratios of Direct-Acting Antiviral (DAA) Initiation
