# Supplementary material for: Association of the Extension for Community Healthcare Outcomes Project With Use of Direct-Acting Antiviral Treatment Among US Adults With Hepatitis C
Source: JAMA Netw Open. 2021 Jul 2;4(7):e2115523. doi: 10.1001/jamanetworkopen.2021.15523 (PMC8254131; doi:10.1001/jamanetworkopen.2021.15523)
Supplement: Supplement. — eAppendix. Discrete-Time Hazard Analysis eTable 1. Status of Extension for Community Healthcare Outcomes (ECHO) Status for Hepatitis C Care by State, 2014-2017 eTable 2. Marginal Effects of Extension for Community Healthcare Outcomes (ECHO) on Direct-Acting Antiviral (DAA) Initiation by Specialist Density Percentile eTable 3. Adjusted Odd Ratios of Direct-Acting Antiviral (DAA) Initiation (Including Patients from New Mexico) eTable 4. Adjusted Odd Ratios of Direct-Acting Antiviral (DAA) Initiation (Including Patients Died within 12 Months from The Index Date*) eTable 5. Adjusted Odd Ratios of Direct-Acting Antiviral (DAA) Initiation [file jamanetwopen-e2115523-s001.pdf]

Supplementary Online Content

Tran L, Feldman R, Riley III T, Jung J. Association of the Extension for Community Healthcare Outcomes project with use of direct-acting antiviral treatment among US adults with hepatitis C. *JAMA Netw Open*. 2021;4(7):e2115523. doi:10.1001/jamanetworkopen.2021.15523

eAppendix. Discrete-Time Hazard Analysis

eTable 1. Status of Extension for Community Healthcare Outcomes (ECHO) Status for Hepatitis C Care by State, 2014-2017

eTable 2. Marginal Effects of Extension for Community Healthcare Outcomes (ECHO) on Direct-Acting Antiviral (DAA) Initiation by Specialist Density Percentile

eTable 3. Adjusted Odd Ratios of Direct-Acting Antiviral (DAA) Initiation (Including Patients from New Mexico)

eTable 4. Adjusted Odd Ratios of Direct-Acting Antiviral (DAA) Initiation (Including Patients Died within 12 Months from The Index Date*)

eTable 5. Adjusted Odd Ratios of Direct-Acting Antiviral (DAA) Initiation

This supplementary material has been provided by the authors to give readers additional information about their work.

© 2021 Tran L et al. *JAMA Network Open.*

eAppendix. Discrete-Time Hazard Analysis

To examine the association between the ECHO program and DAA treatment, we employed a two-way fixed effects difference-in-differences estimator exploiting the variation in exposure to the ECHO program between the treatment and control group. The treatment group includes individuals living in states that have launched ECHO programs since 2006, even if the intervention lasted for a short period (e.g. less than a year), while the control group comprises patients residing in states that never had an ECHO program. We estimate adjusted OR of DAA use for patients in the treatment and control group using a discrete-time hazard model. A similar approach has been used in prior work.[1]

The discrete-time hazard model, utilizing a logistic regression framework, was first proposed by Cox[1] and was studied further by Singer and Willett.[3-7] This approach has become more common in sociology, psychology and education due to several advantages.[8-10] First, this method is well-suited for data that are collected in discrete time intervals, such as longitudinal datasets.[8] Second, this approach allows us to examine how event occurrences vary over time.[7] Third, because this method is specified as a type of logistic model,[2] it is simple and convenient to use. Finally, time-varying covariates can easily be included in the model. However, the discrete-time hazard model has not been widely used in medical research, despite these advantages.

The focus of this model is the hazard of an event, defined as the conditional probability that a single non-repeatable event will occur in a particular time interval, given that the person did not experience the event before that time. As discussed by Willett, Singer, & Martin (1998), the conditionality inherent in the definition of hazard is important because it allows the hazard to "deal evenhandedly with censoring by ensuring that all individuals remain in the risk set until the last time period that they are eligible to experience the event (at which point they are either censored or they experience the target event)."[7]

Because the discrete-time hazard model requires a person-period format, our dataset has a person-year layout with one row for each year of risk. Each row has an indicator for DAA initiation or censoring, which shows if this year of risk ends in the event or not. Once a patient initiates a DAA, he/she is removed from the risk pool and does not contribute any more person-years. Note that no adjustment is needed to account for multiple person-years contributed by a single person. Allison (1982, p. 75) has shown that the likelihood for the discrete-time hazard model is equivalent to that of the logistic regression model with multiple risk periods per person.[11]

The hazard for person *i* in state *s* at year *t* is defined as:

$$P_{ist} = \Pr(t \le T_{is} < t_{+1} \,|\, T_{is} \ge t)$$

T is a discrete random variable indicating the time of occurrence of the event. We estimated the log-odds form of the hazard probability using the following regression:

$$log\left(\frac{P_{ist}}{1-P_{ist}}\right) = \beta_0 + \beta_1 ECHO_{st} + \beta_2 ECHO_{st} * Rural_{ist} + \beta_2 ECHO_{st} * Specialist\ Density_{ist} + \beta_4 State_s + \beta_5 Year_t + \beta X_{ist} + \varepsilon_{ist}$$

where $ECHO_{ist}$ is a measure of the ECHO penetration in state *s* at year *t*
$Rural_{ist}$ is an indicator of ZIP-level rurality of residence of person *i* in state *s* at year *t*
$Specialist\ Density_{ist}$ is county-level specialist density of person *i* in state *s* at year *t*
$State_s$ is a vector of state dummies
$Year_t$ is a vector of year dummies
X_{ist} is vector of demographic and clinical characteristics of person *i* in state *s* at time period *t*

We introduced the discrete-time hazard model at a relatively simple level. Describing more advanced aspect of the model, such as assumptions of linearity, unobserved heterogeneity, and proportionality, is beyond the scope of this paper. Interested readers are referred to previous work by Singer and Willett[3-7] for more technical details.

References

1. Cannonier, C. (2014). Does the family and medical leave act (FMLA) increase fertility behavior?. *Journal of Labor Research*, *35*(2), 105-132.

2. Cox, D. R. (1972). Regression models and life-tables. *Journal of the Royal Statistical Society: Series B (Methodological), 34*(2), 187-202.

3. Singer, J. D., & Willett, J. B. (1993). It's about time: Using discrete-time survival analysis to study duration and the timing of events. *Journal of educational statistics*, *18*(2), 155-195.

© 2021 Tran L et al. *JAMA Network Open.*

4. Singer, J. D., Willett, J. B., & Willett, J. B. (2003). *Applied longitudinal data analysis: Modeling change and event occurrence*. Oxford university press.
5. Willett, J. B., & Singer, J. D. (1993). Investigating onset, cessation, relapse, and recovery: why you should, and how you can, use discrete-time survival analysis to examine event occurrence. *Journal of consulting and clinical psychology*, *61*(6), 952.
6. Willett, J. B., & Singer, J. D. (1995). It's déjà vu all over again: Using multiple-spell discrete-time survival analysis. *Journal of Educational and Behavioral Statistics*, *20*(1), 41-67.
7. Willett, J. B., Singer, J. D., & Martin, N. C. (1998). The design and analysis of longitudinal studies of development and psychopathology. *Development and psychopathology*, *10*, 395-426.
8. Xie, H., McHugo, G., Drake, R., & Sengupta, A. (2003). Using discrete-time survival analysis to examine patterns of remission from substance use disorder among persons with severe mental illness. *Mental Health Services Research*, *5*(1), 55-64.
9. Yabiku, S. T. (2005). The effect of non-family experiences on age of marriage in a setting of rapid social change. *Population Studies*, *59*(3), 339-354.
10. Barber, J. S. (2000). Intergenerational influences on the entry into parenthood: Mothers' preferences for family and nonfamily behavior. *Social forces*, *79*(1), 319-348.
11. Allison, P. D. (1982). Discrete-time methods for the analysis of event histories. *Sociological methodology*, *13*, 61-98.

© 2021 Tran L et al. *JAMA Network Open.*

eTable 1. Status of Extension for Community Healthcare Outcomes (ECHO) Status for Hepatitis C Care by State, 2014-2017

State	Year			
	2014	2015	2016	2017
Alabama				✓
Arkansas	✓	✓	✓	✓
Arizona	✓	✓	✓	✓
California	✓	✓	✓	✓
Colorado	✓	✓	✓	✓
Connecticut	✓	✓	✓	✓
Georgia			✓	✓
Idaho	✓	✓	✓	✓
Illinois	✓	✓	✓	✓
Indiana	✓	✓		✓
Kansas				✓
Louisiana		✓	✓	✓
Maine				✓
Massachusetts		✓	✓	✓
Michigan				✓
Minnesota	✓	✓	✓	✓
Missouri			✓	✓
Montana	✓	✓	✓	✓
New Mexico	✓	✓	✓	✓
New York			✓	✓
North Dakota			✓	✓
Oklahoma		✓	✓	✓
Oregon	✓	✓	✓	✓
Pennsylvania		✓	✓	
South Dakota			✓	✓
Tennessee				✓
Texas	✓	✓	✓	✓
Utah	✓	✓	✓	✓
Washington	✓	✓	✓	✓
West Virginia			✓	✓
Wyoming	✓	✓	✓	✓

© 2021 Tran L et al. *JAMA Network Open.*

eTable 2. Marginal Effects of Extension for Community Healthcare Outcomes (ECHO) on Direct-Acting Antiviral (DAA) Initiation by Specialist Density Percentile

	**dy/dx**	**95% CI**	***p***
Specialist density *(per 1,000 population)*			
1% percentile	1.21	(0.95-1.48)	0.000
25% percentile	1.13	(0.90-1.37)	0.000
50% percentile	1.05	(0.82-1.28)	0.000
75% percentile	0.97	(0.73-1.21)	0.000
99% percentile	0.57	(0.08-1.06)	0.022

© 2021 Tran L et al. *JAMA Network Open.*

eTable 3. Adjusted Odd Ratios of Direct-Acting Antiviral (DAA) Initiation (Including patients from New Mexico)

	**Odd Ratio**	**95% CI**	***p***
**ECHO program** *(100 attendees)*	1.08	(1.07-1.10)	0.000
**Interaction**			
ECHO program × Rural	0.99	(0.98-0.99)	0.000
ECHO program × Specialist density	0.99	(0.99-1.00)	0.015
**Geographic characteristics**			
ZIP-level			
Rural *(ref. Urban)*	1.01	(0.99-1.04)	0.288
County-level			
Specialist density *(per 1,000 population)*	1.00	(0.99-1.02)	0.451
Number of primary care physicians *(county-level)*	0.99	(0.99-1.00)	0.000
State-level			
Total population aged 45 and older *(100,000 persons)*	0.96	(0.95-0.98)	0.000
Total rural population aged 45 and older *(100,000 persons)*	0.90	(0.78-1.04)	0.149
**Female** *(ref. Male)*	0.89	(0.88-0.91)	0.000
**Age**	1.00	(1.00-1.00)	0.152
**Race/ethnicity** *(ref. White)*			
African American	1.54	(1.51-1.57)	0.000
Hispanic	0.90	(0.86-0.95)	0.000
Other	0.78	(0.76-0.82)	0.000
**Clinical comorbidities**			
Cirrhosis	2.08	(2.05-2.3)	0.000
HIV/AIDS	1.08	(1.05-1.12)	0.000
Cancer	0.81	(0.79-0.83)	0.000
Diabetes	0.93	(0.91-0.95)	0.000
Cardiac disease	0.73	(0.71-0.74)	0.000
Eye disease	1.12	(1.09-1.14)	0.000
Bone disease	0.97	(0.95-0.98)	0.000
Kidney disease	0.60	(0.59-0.61)	0.000
Drug and alcohol related disorder	0.70	(0.58-0.61)	0.000
**N** (beneficiaries)	270,174		
**N** (person-years)	406,685		

Abbreviations: ECHO, Extension for Community Healthcare Outcomes; HIV/AIDS, Human immunodeficiency virus/Acquired immunodeficiency syndrome

© 2021 Tran L et al. *JAMA Network Open.*

eTable 4. Adjusted Odd Ratios of Direct-Acting Antiviral (DAA) Initiation (Including Patients Died within 12 Months from The Index Date*)

	**Odd Ratio**	**95% CI**	***p***
**ECHO program** *(whether more than 5 attendees)*	1.09	(1.07-1.11)	0.000
**Interaction**			
ECHO program × Rural	1.01	(0.99-1.03)	0.397
ECHO program × Specialist density	0.99	(0.98-1.00)	0.042
**Geographic characteristics**			
ZIP-level			
Rural *(ref. Urban)*	1.00	(0.97-1.03)	0.814
County-level			
Specialist density *(per 1,000 population)*	1.01	(0.99-1.02)	0.368
Number of primary care physicians *(1,000 persons)*	1.00	(0.99-1.00)	0.000
State-level			
Total population aged 45 and older *(100,000 persons)*	0.96	(0.95-0.97)	0.000
Total rural population aged 45 and older *(100,000 persons)*	0.90	(0.78-1.04)	0.140
**Female** *(ref. Male)*	0.90	(0.88-0.91)	0.000
**Age**	1.00	(1.00-1.00)	0.823
**Race/ethnicity** *(ref. White)*			
African American	1.55	(1.52-1.58)	0.000
Hispanic	0.91	(0.86-0.95)	0.000
Other	0.79	(0.76-0.82)	0.000
**Clinical comorbidities**			
Cirrhosis	2.06	(2.02-2.10)	0.000
HIV/AIDS	1.08	(1.05-1.12)	0.000
Cancer	0.81	(0.79-0.83)	0.000
Diabetes	0.93	(0.91-0.95)	0.000
Cardiac disease	0.73	(0.71-0.74)	0.000
Eye disease	1.12	(1.10-1.14)	0.000
Bone disease	0.97	(0.95-0.99)	0.001
Kidney disease	0.59	(0.58-0.60)	0.000
Drug and alcohol related disorder	0.59	(0.58-0.60)	0.000
**N** (beneficiaries)	278,622		
**N** (person-years)	411,610		

Abbreviations: ECHO, Extension for Community Healthcare Outcomes; HIV/AIDS, Human immunodeficiency virus/Acquired immunodeficiency syndrome
Note: *Index date is the first HCV claim date after a one-year washout period.

© 2021 Tran L et al. *JAMA Network Open.*

eTable 5. Adjusted Odd Ratios of Direct-Acting Antiviral (DAA) Initiation

	**Odd Ratio**	**95% CI**	***p***
**ECHO program** *(whether more than 5 attendees)*	1.01	(0.96-1.06)	0.981
**Interaction**			
ECHO program × Rural	0.99	(0.94-1.04)	0.692
ECHO program × Specialist density	0.97	(0.95-0.99)	0.007
**Years since ECHO implementation**	0.98	(0.95-1.01)	0.241
**Geographic characteristics**			
ZIP-level			
Rural *(ref. Urban)*	1.01	(0.97-1.04)	0.594
County-level			
Specialist density *(per 1,000 population)*	1.01	(1.00-1.03)	0.073
Number of primary care physicians *(1000 persons)*	0.99	(0.99-1.00)	0.000
State-level			
Total population aged 45 and older *(100,000 persons)*	0.99	(0.98-1.00)	0.079
Total rural population aged 45 and older *(100,000 persons)*	0.83	(0.72-0.96)	0.015
**Female** *(ref. Male)*	0.89	(0.88-0.91)	0.000
**Age**	1.00	(1.00-1.00)	0.171
**Race/ethnicity** *(ref. White)*			
African American	1.54	(1.51-1.57)	0.000
Hispanic	0.91	(0.87-0.96)	0.000
Other	0.79	(0.76-0.82)	0.000
**Clinical comorbidities**			
Cirrhosis	2.09	(2.05-2.13)	0.000
HIV/AIDS	1.08	(1.04-1.12)	0.000
Cancer	0.81	(0.79-0.83)	0.000
Diabetes	0.93	(0.91-0.95)	0.000
Cardiac disease	0.73	(0.71-0.74)	0.000
Eye disease	1.12	(1.09-1.14)	0.000
Bone disease	0.97	(0.95-0.98)	0.000
Kidney disease	0.60	(0.59-0.61)	0.000
Drug and alcohol related disorder	0.60	(0.58-0.61)	0.000
**N** (beneficiaries)	267,908		
**N** (person-years)	403,228		

Abbreviations: ECHO, Extension for Community Healthcare Outcomes; HIV/AIDS, Human immunodeficiency virus/Acquired immunodeficiency syndrome

© 2021 Tran L et al. *JAMA Network Open.*
